# A Droplet Generator Using Piezoelectric Ceramics to Impact Metallic Pellets

**DOI:** 10.3390/mi15091139

**Published:** 2024-09-10

**Authors:** Jilong Yu, Daicong Zhang, Wei Guo, Chunhui Jing, Yuan Xiao

**Affiliations:** School of Mechanical & Electronic Engineering, Xi’an Polytechnic University, Xi’an 710048, China

**Keywords:** metallic pellet ejection, piezoelectric ceramic, numerical simulation, ejection generator

## Abstract

Metal micro-droplet ejection technology has attracted attention for its potential applications in the rapid prototyping of micro-metal parts and microelectronic packaging. The current micro-droplet ejection device developed based on this technology faces challenges such as the requirement of a micro-oxygen ejection environment, a complex feeding structure, and high costs. Therefore, a drop-on-demand droplet generator for metallic pellets with impact feed ejection is designed in this paper. This device has a simple and compact structure, does not require a high-cost heat source, and can perform drop-on-demand ejection of metallic pellets in an atmospheric environment. A micro-channel feeding method based on piezoelectric ceramic actuator drives is proposed. A rigid dynamics metallic pellet flight trajectory model is established to analyze the relationships between the driving voltage and the flight trajectory of the pellets. With the help of Fluent to simulate and analyze the melting and ejection processes of the pellets inside the nozzle, the changes in the variable parameters of the flow field in the process of the melting and flight of a single molten drop are studied. The droplet generator produces stable droplets with a 500 µs pulse width and 1100 mm/s initial velocity of the projectile. The simulation results show that a single projectile has to go through three stages including feeding, melting, and ejecting, which take 39.5 ms, 7.85 ms, and 17.65 ms. The total simulation time is 65.0 ms. It is expected that the injection frequency of the metal projectile droplet-generating device will reach 15 Hz.

## 1. Introduction

Molten metal droplet ejection molding technology shows a promising application in the rapid prototyping of micrometal parts [1,2]. This technology overcomes the limitations of traditional rapid prototyping technology in materials, with the advantages of diversified material choices, stable quality of finished products, breakthroughs in the manufacture of complex structures, etc. [3]. Compared with direct laser rapid prototyping technology and three-dimensional micro-welding molding technology, metal melt ejection molding technology has a significant advantage in the manufacture of low-cost, environmentally friendly products [4]. However, the droplets formed due to the breakup of the capillary metal jet ejected from molten metal are influenced by various factors, such as excitation signals, making it challenging to control their shapes, and most require a micro-oxygen ejection environment [5,6]. Based on this, we used the melt spraying method with on-demand feeding of metallic pellets and designed a droplet generator driven by piezoelectric ceramic actuators for metallic pellets.

Metal micro-droplet ejection technology mainly includes continuous uniform droplet ejection and drop-on-demand ejection [7,8,9]. The working principle of continuous uniform droplet ejection is to apply constant pressure in the liquid cavity, forcing the liquid in the cavity to form a jet at a high speed from the nozzle. Then, under the action of the disturbance or surface tension in the fluid cavity, the jet breaks into a droplet of uniform size and stable performance. Finally, through the application of a deflecting electric field [10], the deposition position of the micro-droplet fall is accurately controlled so as to realize the preparation of a variety of three-dimensional structures of the ejection deposition. In the continuous ejection device, it is necessary to pressurize the ejected material and micro-droplet ejection recovery device [11,12], resulting in a complex structure, high cost, and poor controllability of the continuous ejection device. This technology has a wide range of applications in the field of printing large-size advertisements, but it is not mature in the field of direct metal forming. The working principle of metal micro-droplet-on-demand ejection technology [13,14,15,16] is that according to the excitation of the system control signal, when it is necessary to eject micro-droplets, the system gives an excitation signal to the ejection device, and the ejection device produces the corresponding displacement or pressure change after receiving the excitation signal, so as to enable the liquid to be ejected through the small holes to form the required micro-droplets of controllable size. At present, drop-on-demand technology using micro-piezoelectric element actuation has great potential for applications in electronic packaging, line printing, and other areas.

In recent years, Cheng et al. established a pneumatic on-demand injection device [17,18,19,20], that can operate at 573 K and achieve tin injection. A micro-droplet ejection device for metal solder drop-on-demand based on a controllable electromagnetic force drive was developed by Yongxian Wang et al. at Huazhong University of Science and Technology. By controlling the magnitude and direction of the current, thus affecting the spraying, stopping, and size of the droplets, it has good controllability and a fast response. Cao and Miyamoto [21,22,23] developed a net metallic droplet forming technique, netted droplet manufacturing (NDM), which allows the injection of molten aluminum drop-on-demand at 1023 K by using the principle of pneumatic drop-on-demand injection and the forming of simple fabricated parts. Using a self-developed metal micro-droplet pneumatic drop-on-demand ejection device, Fang Ming and other researchers at the University of Toronto, Canada, analyzed the matching relationship among deposition temperature, ejection frequency, and substrate speed for metal micro-droplet deposition when micro-droplets are deposited point-by-point to form a line along the horizontal direction. Qi Lehua et al., from Northwestern Polytechnical University [24,25], developed a controlled spray device for uniform metal microdroplets to realize the spray of tin–lead solder and aluminum alloy, but the device needs to be operated through a glove box in an oxygen-free environment. The use of uniform droplet jet deposition technology in electronics, optics, and biology was investigated and successfully commercialized by Microfab, Inc. (Plano, TX, USA) in the United States. Solder joints for tiny electronic circuits can be printed. In order to explore the application of low-melting-point alloys for electronic packaging, the Composite Materials Center of Beijing Nonferrous Metals Research Institute experimentally investigated the law of metal jet fracture and prepared uniform particles of lead-free solder (Sn-4.0Ag-0.5Cu); for rapid prototyping of micro-metal parts, this droplet generator is able to control droplet sizes and positions accurately, thus realizing the fabrication of high-precision micro-metal structures. This technology has broad application prospects in the fields of micro-sensors, micro-actuators, and microelectromechanical systems (MEMS). For example, in the fabrication of miniature sensors, precision molding of metal parts can significantly improve the sensitivity and stability of sensors. In microelectronic packaging, the droplet generator enables precise metal ball array arrangements for chip packaging and interconnection. Compared with traditional soldering methods, this technology can significantly increase the package density and electrical properties and reduce the package volume; it is suitable for the production of high-density integrated circuits and miniaturized electronic devices. At the same time, by precisely controlling the droplet size and spraying frequency, it can realize an efficient encapsulation process and reduce manufacturing costs.

Aiming at the problems of metal melt droplet equipment, such as the harsh operating environment, complicated process, unstable droplet shape, and complex structure, this paper develops a micro-pressure-driven metal projectile melt droplet generator with a simple structure and integrated feed injection in an atmospheric environment. The Sn40Pb60 metal projectile was used as the printing material to carry out the metal projectile impact feed fusion injection experiment. By means of Workbench rigid dynamics, the flight path of the metal projectile was analyzed, and the relationship between piezoelectric ceramic driving voltage and the flight path of the metal projectile was discussed. The numerical model of metal projectile melt injection was established by using Fluent (2022 R1) software, the process of micro-pressure pneumatic on-demand injection was simulated, and the change in the fluid field in the metal projectile melt injection stage was analyzed.

## 2. Design of the Experimental Setup and Experimental Approach

**Figure 1:**
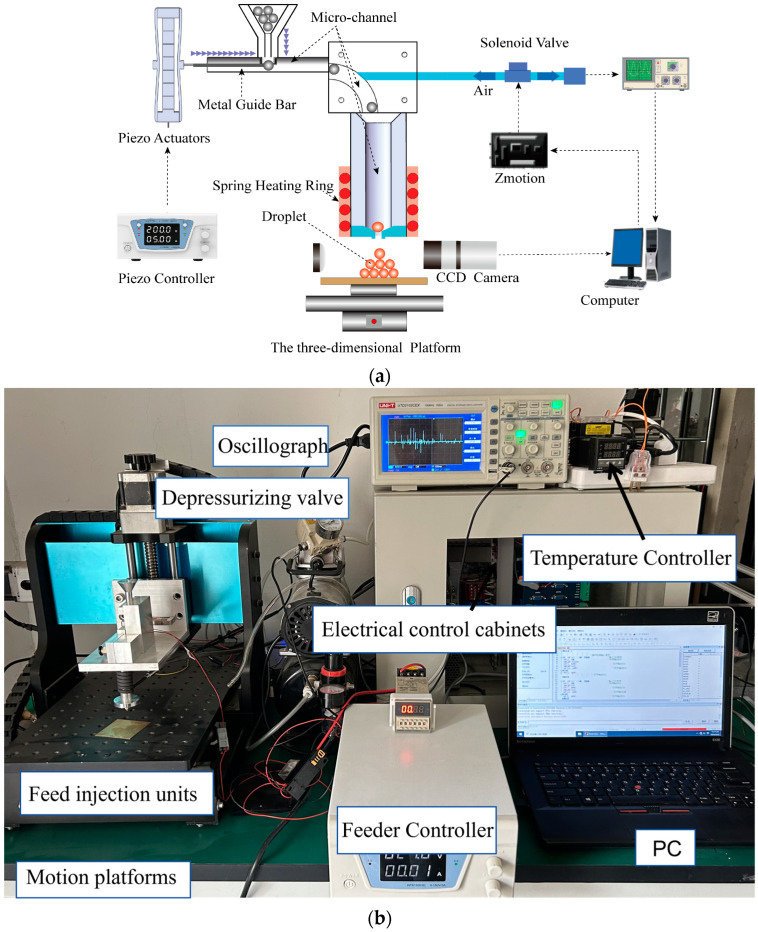
The droplet generation system. (**a**) Schematic diagram of the experimental system. (**b**) Physical picture of the device.

The droplet impact feeding ejection generator feeding ejection process is shown in Figure 1a, and the experimental setup is shown in Figure 1b. This experimental setup is placed in an atmospheric environment. The device is mainly composed of an impact feeding device, a molten droplet spraying device, a pressure data acquisition system, and a CCD high-speed image acquisition system. In order to generate molten micro-droplets, a voltage pulse is first given to the piezoelectric ceramic actuator by the control system, the piezoelectric effect of the piezoelectric ceramics drives the metal guide bar to impact the metallic pellets in the feeding micro-channel, and the metallic pellets are impacted into the micro-droplet ejection channel. The main devices of the molten droplet ejection device are the ejection micro-channel and the spring heating ring; the spring heating ring wraps the ejection micro-channel to heat up and melt the metallic pellets dropped at the nozzle, and in this paper, the temperature of the temperature controller is controlled at 573 K. Air is supplied to the jet micro-channel at one end above the jet micro-channel, and a solenoid valve is connected to the other end, which is switched by the solenoid valve to generate pulse pressure to extrude and deposit molten metallic pellets on the substrate. The working principle of each subsystem is as follows:

### 2.1. Impact Feeding System

**Figure 2:**
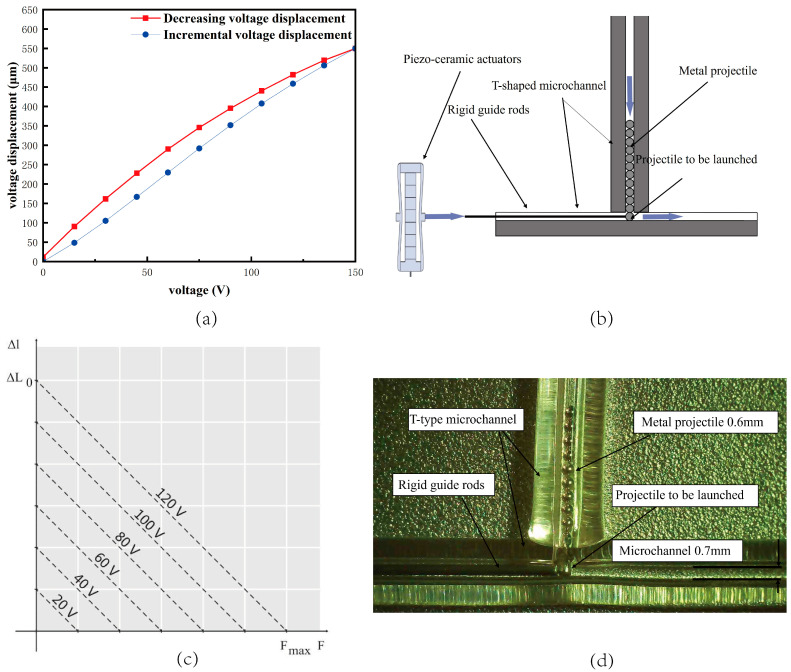
The feeding system. (**a**) Physical piezoceramic actuator and piezoceramic pressure displacement curve. (**b**) Schematic diagram of the feeding micro-channel. (**c**) Piezoelectric ceramic force versus displacement at different voltages. (**d**) T-shaped micro-channel.

The inverse piezoelectric effect of the piezoelectric ceramic increases the output voltage at both ends of the ceramic, causing it to deform. The ceramic strain is very small, so its deformation is only one-thousandth of its length. The piezoelectric ceramic actuator’s displacement is the primary index of selection. The displacement output direction and size differ; the shorter the piezoelectric ceramic displacement, the better the feeding device’s machining and assembly accuracy. As a result, the output displacement is the primary selection index. In this paper, Harbin Core Tomorrow Technology is used produce the PZT cobalt titanate lead low-pressure co-fired piezoelectric stacked ceramic displacement amplifier. The displacement of the piezoelectric ceramic is proportional to the applied voltage, and the maximum displacement of the piezoelectric ceramic displacement amplifier is 550 µm under the full load voltage of 150 V. The piezoelectric ceramic circuit is a simple voltage pulse circuit, and the frequency of the actuator is controlled by adjusting the frequency of the voltage pulses. As shown in Figure 2a, the red color is the incremental voltage, and the voltage is stepped in steps of 15 V sequentially from 0 V until the loaded voltage is 150 V. So far, the voltage has decreased from 150 V to 15 V sequentially, and the step voltage has decreased to 0 V. The incremental voltage and decremental voltage among the output displacement differences are the hysteresis characteristics of piezoelectric ceramics. As can be seen in the figure, piezoelectric ceramics from the 0 V loaded voltage to 150 V full load process roughly follow the linear change rule. From 150 V full load start unloading voltage, the ceramic at each voltage of the displacement compared with the loading of the corresponding voltage displacement shows a hysteresis change, with displacement hysteresis from small to large. In the 45–75 V region, the displacement hysteresis reaches its maximum value; below 45 V, the hysteresis slowly becomes smaller, until unloaded to the initial voltage of 0 V, when the displacement hysteresis is the minimum, almost close to zero. Therefore, in this paper, when choosing the applied voltage, we pay attention to avoiding voltage intervals with large hysteresis. Under full load, the displacement amplification structure sends out a 500 µm displacement. The impulse that is created at this moment goes through the metal guide rod, and the stored energy is transferred to the guide rod through deformation. This causes the guide rod to strike the metallic pellets, giving the pellets their initial speed and starting the feeding process.

The feeding system’s main function is to feed metallic pellets into the jetting microchannel continuously. The manufacturing process of the microchannel glass tubes involves first prefabricating them to the correct size. The raw material, prefabricated quartz glass, is placed into a tube-drawing furnace equipped with a precise temperature control system to ensure uniform softening of the glass by gradually increasing the furnace temperature to the softening temperature of quartz glass. After softening, the required diameter of the blank tube is drawn by precisely controlling the drawing speed and force to ensure uniform wall thickness and smooth internal and external surfaces. Based on the drawn blank tube, it is cut using high-precision cutting tools to obtain the required length and initial shape. Finally, the blank tube is finely processed using laser-melting technology to form a microchannel. In this paper, the selected metallic pellets have a material diameter of 0.6 mm. In order to be able to feed nearly frictionless metallic pellets into the jet channel accurately, the feeding channel using the capillary quartz channel effectively reduces the friction. The capillary quartz channel is inverted into a “T” shape. The piezoelectric ceramic actuator applied voltage, driven by the metal guide bar, impact is located in the horizontal capillary quartz channel launch position of the metal pellets. When the launch position of the metal pellets is knocked away, vertically arranged in the T-shaped quartz channel in the metal pellets under the force of gravity, downward displacement is made to the launch position to wait for the next impact action, as shown in Figure 2c. The launched metallic pellets fall down to the nozzle along the feed microchannel under the action of impulse energy and reach the melt injection part.

### 2.2. Molten Droplet Ejection Device

The device for ejecting molten droplets includes spring heating rings, thermocouples, temperature controllers, jet micro-channels, brass nozzles, and other devices. The spring heating ring heats up the jet micro-channel, and under the action of high temperature, the metallic pellets at the tip of the nozzle are melted in time and then sprayed out of the nozzle under the action of micro-pneumatic pressure. A temperature that is too high can cause denaturation in the material, which can impact the jetting results. Conversely, a temperature that is too low can influence the melt molding rate. The thermocouple, which provides feedback to the temperature controller, controls the nozzle temperature. In this paper, we set the temperature controller to 573 K.

### 2.3. Data Acquisition System

The main devices of the pressure data acquisition system are pressure sensors, oscilloscopes, and signal conversion modules. The pressure sensor is installed at one end of the pressurized channel to collect and record the pressure fluctuation. The measurement range of the pressure sensor used in this device is 0–0.25 MPa, the signal from the pressure sensor is converted into a voltage signal by the signal conversion module, and the data are recorded by an oscilloscope (UTD2000, UNI-TREND TECHNOLOGY, Dongguan, China). The CCD high-speed image acquisition system consists of a CCD high-speed camera (i-SPEED 220,Beijing Yanxintong Technology Co., Ltd., Beijing, China), a microscope (K2 DistaMax, Infinity, Centennial, CO, USA), and a strobe-free LED light source (Danny U-100T, Beijing, China). The system works by taking a single image with a CCD camera as a drop of molten metal is ejected from the nozzle, while a solenoid valve is turned on and off once and an oscilloscope records the pressure data once. Since a single drop can be generated many times over, by delaying the trigger time of the CCD camera, the drop generation process can be recorded sequentially.

## 3. Simulation

### 3.1. Simulation of Rigid Dynamics

#### 3.1.1. Mathematical Model

Metallic pellets do not change their shape during flight, and their flight process can be studied using rigid dynamics. Numerical simulation of the flight simulation of metallic pellets by solving rigid dynamics equations can accurately consider the kinetic effects of metallic pellets during flight.

In the calculation, the piezoelectric ceramic creep characteristics cannot be ignored [26,27], that is, when applying voltage to the piezoelectric ceramic, the piezoelectric ceramic will be immediately followed by elongation to produce the corresponding displacement. In the subsequent long period of time, the piezoelectric ceramic length will continue to produce a small elongation deformation, which will be followed by the small length of the deformation. The amount of creep is calculated in the following Formula (1).

(1) $$\Delta l(t)=\Delta l_{0.1}\left(1+\gamma \cdot \mathrm{lg}\cdot \frac{t}{0.1s}\right)$$

where ∆*l(t)* is the piezoelectric ceramic displacement; ∆*l*_0.1_ is the displacement after applying voltage for 0.1 s; and *γ* is the creep coefficient, which generally takes the value of 1~2%.

The piezoelectric ceramic step time can be understood as the fastest response time of the piezoelectric ceramic. When the piezoelectric ceramic is loaded with a very fast voltage signal, the piezoelectric ceramic will be in the step time of rapid expansion and deformation, and, at the same time, the piezoelectric ceramic resonance frequency will be provoked, resulting in a damping oscillation to reach a stable position; the piezoelectric ceramic rapid expansion and deformation of the shortest rise time is determined by its resonance frequency. When the control voltage signal is rapidly increased to the maximum, the piezoelectric ceramic will be at a resonant frequency of one-third cycle to reach the maximum value of the displacement. This paper uses PZT cobalt lead titanate low-pressure co-fired piezoelectric stacked ceramics at a no-load resonant frequency of 260 Hz, with a step time of 1282 μs.

The piezoelectric ceramic output force is generated by the compression loss of output displacement, which is obtained by detecting the displacement loss of piezoelectric ceramics, and there is a linear relationship between the change in force and the compensation voltage of the core tomorrow R500, which is shown in Figure 2c and Equation (2). In the voltage excitation of 150 V, the outgoing force is 6000 N. The relationship between the initial flight velocity of metallic pellets and the piezoelectric ceramic force can be calculated by the following equation. Different applied voltages correspond to different piezoelectric ceramic forces, so, combined with Equations (2) and (3), different voltages applied by the forces can be used to apply the corresponding initial flight velocity to the metallic pellets.

(2) $$k_{A}=\frac{F_{\max}}{\Delta L_{0}}$$

where *F_max_* is the maximum output force and ∆*L*_0_ is the output displacement.

The change in impulse of a metallic pellet’s flight under force can be expressed by the following equation:

(3) $$\left\{\begin{array}{l} F_{x}\cdot \Delta t=m\cdot \Delta v_{x}\\ F_{y}\cdot \Delta t=m\cdot \Delta v_{y}\\ F_{z}\cdot \Delta t=m\cdot \Delta v_{z} \end{array}\right.$$

where *F* is the force applied to the metallic pellet, Δt is the time at which the force is applied to the metallic pellet, i.e., the step time of the piezoelectric ceramic, and *m* is the mass of the metallic pellet.

The change in position and attitude of rigid dynamics in space can be described by the equations of dynamics of the center of mass advection of rigid dynamics in an inertial system:

(4) $$\left\{\begin{array}{l} m\frac{dV_{x}}{dt}=F_{x}\\ m\frac{dV_{y}}{dt}=F_{y}\\ m\frac{dV_{z}}{dt}=F_{z} \end{array}\right.$$

where *m* denotes the mass of the rigid body, *V_x_*, *V_y_*, and *V_z_* denote the velocity of the rigid body along the three coordinate directions of *x*, *y*, and *z*, and *F_x_*, *F_y_*, and *F_z_* denote the force on the rigid body in the three coordinate directions.

**Figure 3:**
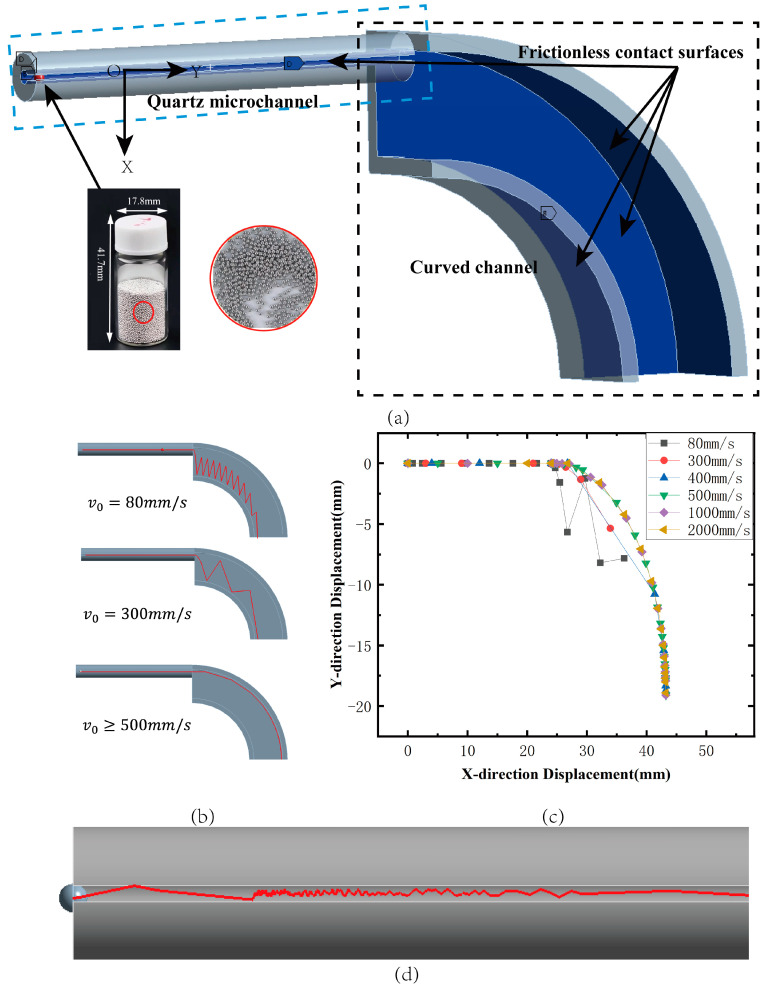
Simulation of the projectile flight trajectory during feeding. (**a**) Modeling of the feed flight trajectory and contact setup. (**b**) Flight trajectory of a metal projectile at different initial velocities. (**c**) Plots of flight trajectories at different initial velocities. (**d**) Advancing trajectory of the projectile in the case of an uncentered collision.

Workbench rigid dynamics is used to simulate the process of metal pellets being impacted and flying. As shown in Figure 3a, the inner diameter of the feed microchannel is 0.7 mm, and the diameter of the metal pellet particles is 0.6 mm. According to the actual situation, in order to improve the simulation efficiency, the following assumptions are made for the simulation model: (1) the friction between the metallic pellets and the microchannel is negligible; (2) the impact of the metal guide bar on the pellets is a central impact; based on the above conditions, the motion of the metal pellets in the feed microchannel can be simplified to a parabolic motion. The contact conditions of the simulation model are shown in Figure 3a, and the contact conditions of the simulation model are shown in Figure 3b. The impact is a centripetal collision; based on the above conditions, the trajectory of the metallic pellets in the feeding microchannel can be simplified as a parabolic motion. The contact conditions of the simulation model are shown in Figure 3a, where the model retains the entities that come into contact with the pellets during their flight. The contact between a total of four surfaces of the inner wall of the quartz microchannel and the curved channel and the spherical surface of the metallic pellets is set to be frictionless. The curved channel is fixedly connected to the ground, the contact surfaces between the two entities are set as no-slip (welded joints), and standard earth gravity g = −9806.6 mm/s^2^ is applied globally.

#### 3.1.2. Simulation Analysis

The initial velocity of the pellet is mainly affected by the applied voltage of the piezoelectric ceramic actuator, and the flight trajectories of metallic pellets under different initial velocities can be observed through rigid dynamics simulation (shown in Figure 3b,c). When the initial velocity of the metal ball is low (80 mm/s), the motion of the ball in the pipe is mainly affected by gravity and collisions with the inner wall of the pipe. Because of the slower horizontal velocity, the ball accelerates more in the vertical direction, leading to its rapid descent and elastic collision with the inner wall of the pipe. This bouncing effect leads to the phenomenon of fluctuation in the trajectory. When the initial velocity is larger (500 mm/s and above), the inertia effect of the ball is stronger in the horizontal direction, and the centrifugal force causes it to cling to the inner wall of the pipe, which reduces the frequency of collision and bouncing. As a result, the trajectory of the ball is smoother. Through these two different motion behaviors, we can conclude that the larger the initial velocity, the smoother the trajectory of the ball, which is due to the combined effect of inertia and centrifugal force. This makes the ball reduce the frequency of collision with the inner wall of the pipe, thus avoiding the fluctuation phenomenon. In addition, the simulation found that when the metal guide bar and metallic pellets do not collide centripetally, as shown in Figure 3d, they constantly collide up and down in the pipe and show irregular movement, which leads to the loss of control of the feeding time, so attention should be paid to the accuracy of the installation during the physical installation to avoid the occurrence of this phenomenon. Combined with Formulas (1)–(4) that were obtained in this study using piezoelectric ceramic, the maximum displacement (i.e., the minimum force) can be applied to metallic pellets with an initial velocity of 1100 mm/s. According to the simulation results, this initial velocity feeding mechanism requires 39.5 ms to complete the feeding, and the feeding frequency can be achieved at 25 Hz.

### 3.2. Simulation of Fluid Dynamics

#### 3.2.1. Physical Model

In order to verify the feasibility and accuracy of the developed metallic pellet ejection device, this paper used Sn40Pb60 alloy particles with a diameter of 600 µm as the ejection material for the metallic pellet ejection test (material properties are shown in Table 1). By observing the morphology of tin–lead alloy molten droplet particles produced by the ejection device at different temperatures, the range of spring heating coil heating temperatures that can realize micro-droplet ejection in a stable manner was determined. The feasibility of the micro-droplet ejection system was verified by adjusting parameters such as the air supply pressure and driving voltage in order to achieve uniform molten metal droplet ejection.

With the help of Fluent (2022 R1) computational fluid dynamics software, the process of metallic pellet feeding and the gas inside the injection channel at a given air pressure were simulated using the finite volume method (VOF). Based on the measured nozzle, the geometric model was established using Design Modeler (2022 R1) software, and then the mesh grid was divided; the mesh grid file was read into Fluent software and, finally, the boundary conditions and material properties of the model were set in Fluent. Metallic pellets melt the process of volume change, and the calculation of the pressure field belongs to a typical adiabatic, unpressurized, unsteady fluid flow process. In this paper, in the droplet generation area, we established a cylindrical coordinate (*Z*, *r*, θ) system, where *Z* is the axial coordinate, *r* is the radial coordinate, θ is the azimuthal coordinate, the origin of the cylindrical coordinate. The system is set on the jet axis, and the nozzle outside the plane of the intersection of the *Z*-axis in the direction of the force of gravity was in the opposite direction. The inside of the nozzle is set in the direction of gravity, and the nozzle was set in the direction of gravity. The direction is opposite to that of gravity, and the inside of the nozzle is set as a no-slip wall boundary condition.

**Figure 4:**
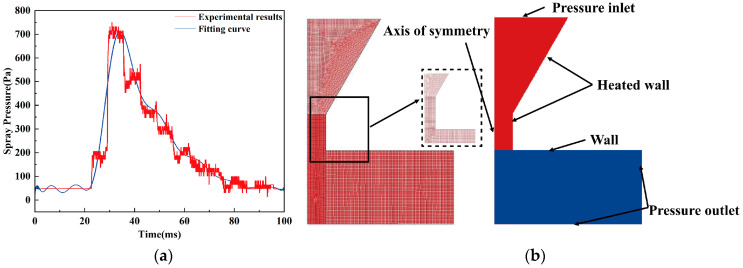
Boundary conditions for fluid simulation. (**a**) Pressure fluctuation fitting curve. (**b**) Boundary conditions of the model.

As shown in Figure 4b, the upper part of the computational region is the pressure inlet boundary condition. Considering that the gas pressure data directly measured in the test are not easily loaded directly as the boundary condition of the simulation model, the measured pressure pulse waveform can be characterized by fitting the measured data with a Fourier series, i.e., a functional expression for the pressure pulse expressed as the sum of multiple sinusoidal and cosinusoidal perturbations of different frequencies. This paper adopts the m-order Fourier series. The test data are fitted, and the fitted function as the pressure inlet function is loaded into the model. As shown in Figure 4a, where the black line is the experimental data and the red line is the fitted curve, the waveform of the fitted function matches well with the experimental data, so the fitted function as the pressure inlet function can effectively respond to the impulse pressure of the liquid surface in the actual injection process. The lower part of the calculation area is the pressure outlet boundary condition.

(5) $$P(t)=A_{0}+\sum_{n=1}^{m}A_{n}\cos2n\pi ft+\sum_{n=1}^{m}B_{n}\sin2n\pi ft$$

where $A_{n},B_{n}$ are the fitting coefficients and f is the pulse frequency, which can be calculated from pressure pulse measurements, and *m* is the Fourier series order. For barometric pulse pressure fluctuations, *m* is generally taken as 15.

The boundary conditions are set schematically, as shown in Figure 4b, with a grid density of 0.005 mm, a nozzle diameter of 0.3 mm at the maximum, a minimum of 0.6 mm, and an orifice height of 2 mm. The axis of symmetry is taken as the axis of symmetry between the nozzle axis and the right edge of the gas zone. The nozzle surface is set as a standard wall, and the pressure inlet is considered to be fixed for scalar-type variables such as total pressure on the inlet boundary. The pressure inlet is the pressure inlet, the pressure inlet function is loaded, the heated wall simulates the heated portion of the nozzle, and the temperature is set to 590 K. The cell marking function is used to determine the position of the metallic pellets, and the area is set to be fluid. In order to show the position of the pellet, the initial temperature of the pellet is set to be slightly higher than the temperature of the air, and the temperature of the air is set to be slightly higher than the temperature of the pellet. The fluid area of all nozzle sections is a unit group, and the information input to the fluid area is the type of fluid medium (material).

According to experimental reality, in order to improve the simulation efficiency, this paper makes the following assumptions before the simulation: (1) The density of the material is set to ideal gas. (2) In the viscous flow model, the no-slip boundary condition is defaulted at the wall [25]. (3) Turbulence inside the nozzle and droplets is ignored. (4) The molten droplet generation process is an adiabatic, non-pressurizable, unsteady fluid flow process.

Therefore, in this paper, the continuity equation of fluid flow and the mass conservation equation are used in the calculations:

(6) ∇⋅V=0

The hydrodynamic conservation equations, i.e., the Navier–Stokes equations, are as follows:

(7) $$\frac{\partial V}{\partial t}+\nabla \cdot (VV)=-\frac{1}{\rho_{l}}\nabla p+\frac{\mu }{\rho_{l}}\nabla \cdot (\nabla V+\nabla V^{T})+g+\frac{1}{\rho_{l}}F_{\mathrm{vol}}$$

where *V* is the velocity vector, *g* is the gravity vector, *p* is the pressure vector, *t* is time, and *ρ_l_* and *μ* are the density and viscosity of the fluid, respectively. Once again, in the model, surface tension is viewed as a continuous, three-dimensionally acting localized volumetric force (*F_vol_*) on a free surface. *F_vol_* can be expressed as:

(8) $$F_{vol}=\rho_{l}\frac{\bar{\rho }k_{l}\nabla a_{l}}{0.5(\rho_{l}+\rho_{g})}$$

where *k_l_* is the slope of the surface at the localized air-pressure interface. The scalar function *a_l_* is defined as the droplet volume fraction in the cell grid in the computational region, which is used to track the gas–liquid interface. The function can be expressed as:

(9) $$\frac{\partial a_{l}}{\partial t}+v\cdot \nabla a_{l}=0$$

In Equation (8), $\bar{\rho }$ is the average density of the fluid in the cell where the free surface is located, and this average density can be defined as:

(10) $$\bar{\rho }=a_{l}\rho_{l}+(1-a_{l})\rho_{g}$$

The effect of surface tension on the free surface flow is obtained by calculating Equations (7)–(9), after which it can be solved by the momentum conservation equation expressed in Equation (6).

The ejection material is Sn40Pb60 with a liquid phase line temperature of 463 K. The parameters such as alloy density, surface tension, and fluid viscosity can be calculated from the following equation at a temperature *T* (*T* is greater than the liquid phase line temperature):

(11) $$X=X_{m}+\left(T-T_{m}\right)(dX/dT)$$

where *X* and *X_m_* are the parameters of density, surface tension, and fluid viscosity of the alloy at injection temperature *T* and liquid phase line temperature *T_m_*, respectively; the parameter *dX*/*dT* is the rate of change in the corresponding parameters with different temperatures. In order to ensure that the metallic pellets are completely melted at the time of ejection, the temperature of the heated wall is set to 590 K.

#### 3.2.2. Simulation Analysis

**Figure 5:**
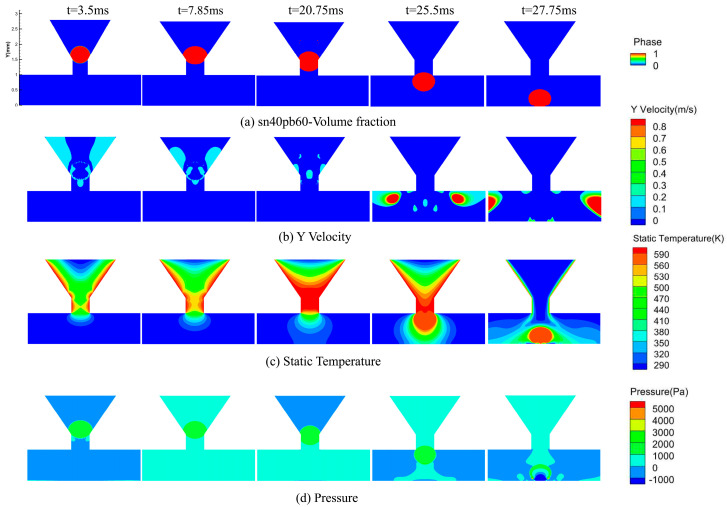
Volume fraction, velocity, temperature, and pressure clouds of molten metal droplets.

According to the above pre-processing part of the simulation solution for the melting and ejection process of tin–lead metallic pellets, the changes in the phase, pressure, and velocity fields around the nozzle during the ejection process are monitored. The following figure shows the axial velocity distribution of the fluid domain and the droplets, the pressure distribution, and the change in the profile of the droplets before and after the generation of the metallic droplets. Figure 5a confirms that metallic pellets undergo three phases of “melt–eject–fly” at the nozzle under a single pressure pulse. At 3.5 ms, the shape of the ball starts to change; at this time, the part of the ball in contact with the heated nozzle wall starts to melt, and the air at the contact point of the ball is heated and flows, resulting in the *Y*-axis axial air flow. At 7.8 ms, the ball is completely melted, and it starts to enter the nozzle orifice under the action of the inlet pressure until 25.5 ms, when the ball is completely detached from the orifice. In the process, the ball experiences an up-and-down oscillation. In Figure 5d, we can observe that the pressure below the ball is greater than the pressure above the ball at 20.75 ms, which is the reason for the oscillation of the ball, and the ball enters the air domain and starts to fly after 25.5 ms. The temperature of the air domain inside the nozzle gradually decreases, but the heated wall is always set to 590 K, and the ball flies downward with the heat.

##### Projectile Melts

**Figure 6:**
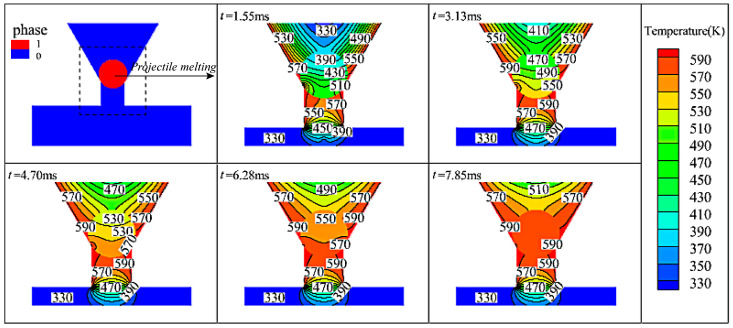
Isotherm of temperature distribution during melting of metal particles in the nozzle.

As shown in Figure 6, the metallic pellets are in a solid–liquid phase transition between 0 and 7.85 ms, and the pressure tends to decrease gradually from the center of the orifice to both sides of the nozzle, as shown in Figure 5d. Heat is gradually transferred to the interior of the metallic pellets from the contact point between the pellet and the heated wall as the heating time becomes longer. The morphology above the contact point line changes from circular to elliptical when the metallic pellets melt (Figure 5a), and the temperature of the fluid domain inside the nozzle is gradually warmed up to 590 K. The simulation shows that a single pellet takes 7.85 ms to convert from the solid state to the liquid state.

When the melting was completed at 7.85 ms, the pressure inlet boundary condition forced the metal droplets to start to extrude the jet hole, and the droplet morphology changed because of extrusion. At 25.5 ms, the droplets completely separated from the nozzle and entered the air domain. As can be seen from the temperature cloud map in Figure 5c, the heat carried by the droplets gradually moves downward as the droplets move during ejection. The velocity cloud diagram in Figure 5b shows that the axial velocity at the nozzle decreases during ejection, which is due to the reduction in droplet kinetic energy because of the work performed by overcoming the pressure of the inner wall of the nozzle during ejection. The pressure cloud map in Figure 5d shows that the pressure inside the fluid domain is less than 1 kPa and evenly distributed, which is the main reason why the shape of the droplets does not change much during the ejection process. The simulation results of the morphology map show that the morphology changes little before and after the formation of droplets during the ejection process, which is conducive to the formation of spherical metal particles. After melting, the metal droplets were ejected from the nozzle, and it took 17.65 ms to fully enter the air domain. The feeding, melting, and spraying of a single projectile took 39.5 ms, 7.85 ms, and 17.65 ms, respectively. The overall simulation time was 65.0 ms, and the injection frequency could reach 15 Hz.

**Figure 7:**
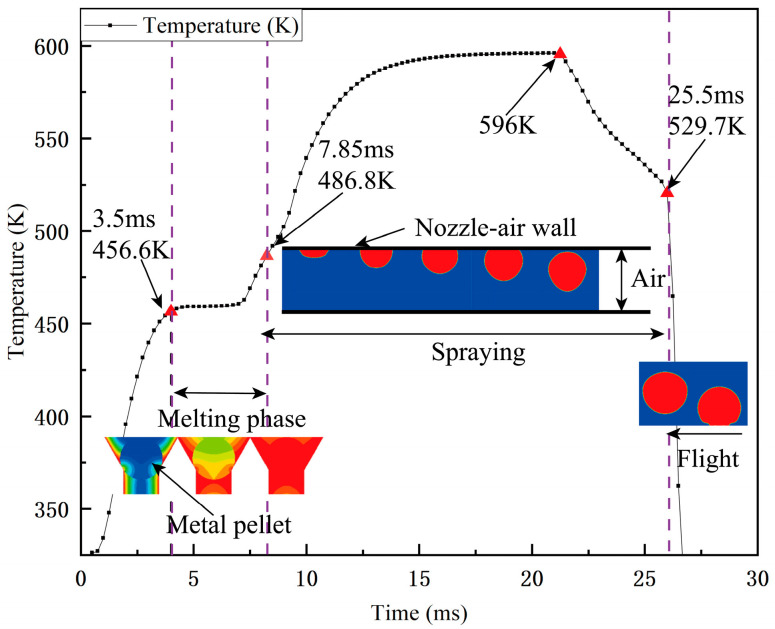
Heat transfer and morphology of molten metal droplets in the melt–ejection stage.

In order to further understand the temperature changes at each stage of droplet molding, a probe was added to the center of the ball at the initial position of the ball to collect the temperature data at this point, as shown in Figure 7. Combined with the phase diagrams, it can be seen that the temperature at this point rises from the initial temperature to 456.6 K in 3.5 ms, the metal ball begins to melt at the point of contact with the nozzle in 3.5 ms, and the metal ball is completely melted in 4.35 ms. The heat is transferred from the heating wall to the metal ball, and the temperature at this point rises slowly to 486.8 K. The droplet reveals s curved moon surface at 7.85 ms, and more and more fluid exits the flow under the continuous pressure inside the nozzle. Then, the metal droplet with the shape of an approximate sphere enters the air domain completely away from the nozzle at 25.5 ms, and the temperature reaches the maximum value of 596 K. With the droplet’s descent, the heat is taken away by the droplet, the temperature at the probe rapidly decreases, and the metal ball enters the flight stage.

In addition, with the help of the simulation model, it can be observed that the molten metal droplets oscillate up and down with a small amplitude during the ejection phase. It can be seen from the results of the simulation volume fraction that the molten metal droplets start to oscillate at 12.25 ms, stop oscillating at 14.75 ms, and then continue to fall. From the pressure cloud diagram (d), it can be seen that the external pressure is slightly larger than the nozzle air pressure during this time. The pressure difference between the external pressure and the inside of the nozzle is formed, and as the pressure in the pressure inlet increases, the molten drop continues to fall. Therefore, the phenomenon of up-and-down oscillation of the molten droplet is formed, which is also the reason why the molten droplet is flattened in the process of falling.

## 4. Experiment and Discussion

According to the simulation results, the stabilized spraying process parameters of metal molten droplets, as shown in Table 2, were established and applied to the molten spraying experimental test of Sn40Pb60 metal metallic pellets. The piezoelectric ceramic driving voltage was 120 V, the vibration frequency was 15 Hz, and the supplied air pressure was 800 Pa. The pulse width of 500 µs could stabilize the production of a single molten droplet of the tin–lead alloy.

**Figure 8:**
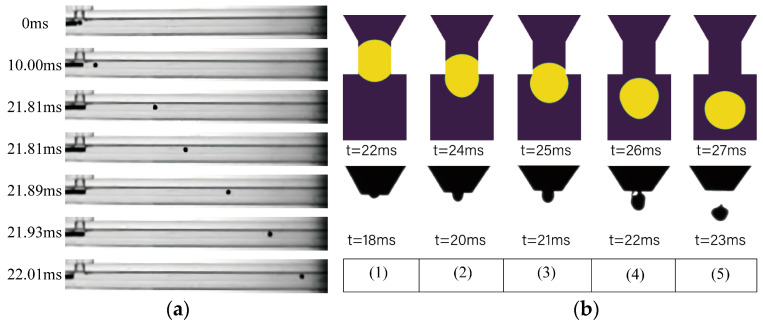
CCD experimental plots (**a**) Feeding: variation in the position of the spheres in the T-type microchannel. (**b**) Ejection: generation time of the droplets under pulsed pneumatic pressure.

A piezoelectric ceramic drive was used to carry out feeding experiments. By monitoring the impact of the ball and the movement of the ball in the T-type microchannel using the strobe exposure method, with the shooting process of the CCD camera shutter set to the slowest stop through the time-delayed flash, we captured the position of the metal pellets at different moments. The results of the experiment are shown in Figure 8a. From the launch position, the time consumed for the metal pellets to fly out of the T-type microchannel was 22.01 ms. the experimental results and the simulation results differ from the simulation results of 0.12 ms. Combined with the simulation time for the process of entering the curved channel and reaching the nozzle, the whole feeding process takes between 39.5 and 39.62 ms.

The pressure pulse shown in Figure 1b is used to simulate the molten droplet ejection process and the metallic pellet molten ejection test. The test process is shot using the strobe exposure method, and the shutter of the CCD camera in the shooting process is set to the slowest stop. Through the time-delayed flash, the metal molten droplets can be captured at different moments in the flight image. The numerical simulation results and captured test results are shown in Figure 8b below. A scimitar crescent surface appeared 4 ms later than the simulation results, and the entire complete spray time was also 4 ms later than the simulation time. The reason may be due to the simulation condition error, the spray trigger information demonstration, and other factors. The average diameter of the molten droplet obtained from the simulation results and the average diameter of the molten droplet obtained from the shooting are 0.6 mm and 0.57 mm, respectively, and the results are more in line with each other.

**Figure 9:**
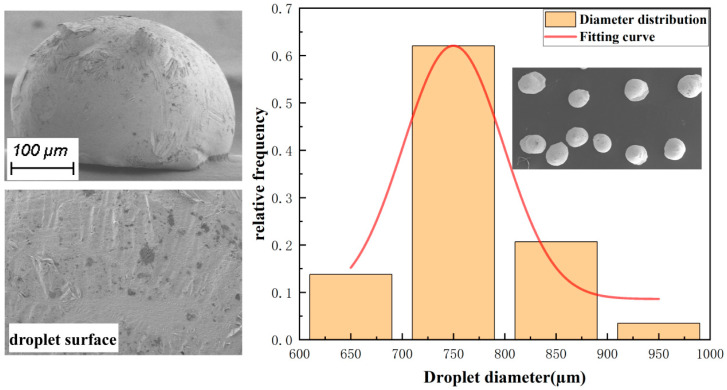
Droplet size distribution.

Taking tin–lead particles as the experimental object, the molten droplets obtained by depositing molten tin–lead particles under the process parameters are shown in Table 2, and the photographs obtained by enlarging and photographing through GeminiSEM 360 SEM are shown in Figure 9. The results show that the micro-droplets are deposited in contact with the substrate to form particles with the morphology of a spherical crown.

**Figure 10:**
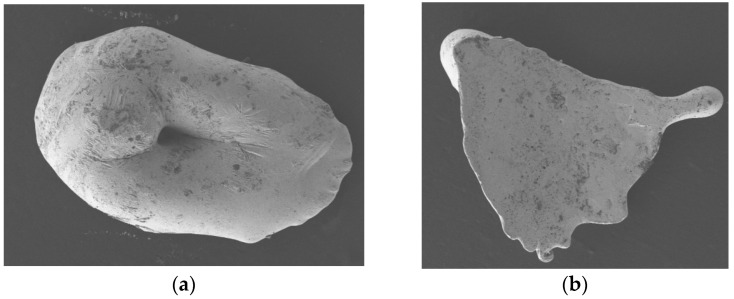
Other shaped metal droplets. (**a**) Multiple melt droplets fused to form a large melt droplet. (**b**) Unstable air pressure working to produce flakes.

The ejection experiments show the initial stage of metal droplet spraying and the long time required when the spraying effect is poor. In the initial three to six pulses, metallic pellets easily accumulate a block at the nozzle. After melting, as shown in Figure 10a, they are sprayed as composite large molten droplets, which may be due to the setup of the spring heating coil. The beginning of the heating is insufficient, leading to the projectile not being completely melted. Its diameter is slightly larger than the diameter of the nozzle, and it cannot be sprayed by micro-pneumatic pressure, therefore causing the formation of clogging. In addition, the test found that this device, when used for a long period of time, will have spray instability. The reason may be due to the solenoid valve not being regularly opened and closed, resulting in an instantaneous increase in the pressure in the micro-channel so that the sprayed molten droplets were, as shown in Figure 10b, pressed into flakes.

The obtained droplet diameter data were imported into the data processing software (Origin 2022), and the average diameter of the droplets was found to be 759 µm. The standard deviation of the metal droplets was 71.207 µm (large droplets and flaky droplets produced by instability were not counted), which is attributed to the presence of large multiple fused droplets at the beginning of the device, proving the above analysis. Therefore, in order to ensure printing accuracy, the heating unit needs to be turned on in advance so that the nozzles and microchannels are sufficiently preheated. Furthermore, the device should not be operated for a long period of time under the current experimental conditions.

## 5. Conclusions

In this paper, the physical and fluid field parameters of metal particle feeding, melt, and spray forming processes are investigated with the help of rigid dynamics and the Fluent modules of Workbench (2022 R1) software. Based on this, a drop-on-demand technique for a metal particle piezoelectric ceramic feeding–melt–jet drop generator is designed. The main conclusions are as follows:

(1) A metal particle piezoelectric ceramic feed–melt–jet droplet generator was designed, which goes through the following three stages: feeding, melting, and ejection. The droplet generator produces stable droplets with a pulse width of 500 µs and an initial velocity of 1100 mm/s of the projectile.
(2) A rigid dynamics metal pellet feeding flight trajectory simulation based on the finite volume method and a micro-pneumatic-type metal pellet spraying process simulation model were established.
(3) The initial velocity of the metal pellet flight is 1100 mm/s, and the simulation of the feeding, melting, and ejecting process of the metal pellet feed takes 39.5 ms, 7.85 ms, and 17.65 ms, respectively, with a total time of 65.0 ms. The theoretical ejection frequency of the device reaches 15 Hz.

## Figures and Tables

**Table 1:** Thermo-physical properties of Sn40Pb60 [28].

Properties	Value
Density (kg·m^−3^)	$\rho_{d}\;$= 8500
Surface tension (N·m^−1^)	$\sigma_{d}=0.494$
Dynamic viscosity (Pa·s)	$u_{d}=0.00135$
Heat conductivity coefficient (W·m^−1^K^−1^)	$k_{\mathrm{liquid}}=30,\;k_{\mathrm{solid}}=49$
Specific heat (J·kg^−1^K^−1^)	$C_{d\;}=186.2$
Latent heat of fusion (J·kg^−1^)	$H_{d\;}=47,560$
Liquidus temperature (K)	$T_{\mathrm{liquid}}=463$
Solidus temperature (K)	$T_{\mathrm{solid}}=456$

**Table 2:** Experimental process parameters for tin-lead molten drop deposition.

Particle Diameter (µm)	Nozzle Diameter (µm)	Pulse Width (µs)	Heating Temperature (K)	Supply Pressure (Pa)	Deposition Distance (mm)
600	500	500	590 K	800	15

## Data Availability

The data that support the findings of this study are available from the corresponding author upon reasonable request.
